# Exosomal circPABPC1 promotes colorectal cancer liver metastases by regulating HMGA2 in the nucleus and BMP4/ADAM19 in the cytoplasm

**DOI:** 10.1038/s41420-022-01124-z

**Published:** 2022-07-23

**Authors:** Yang Li, Jialei Hu, Meng Wang, Yihang Yuan, Fangyuan Zhou, Haosen Zhao, Tianming Qiu, Leilei Liang

**Affiliations:** 1 Department of Laboratory Medicine, the Affiliated Hospital of Jinzhou Medical University, 121001 Jinzhou, China; 2 Clinical Laboratory, Yiwu Maternal and Child Health Hospital, 321000 Jinhua, China; 3 National Cancer Center/National Clinical Research Center for Cancer/Cancer Hospital, Chinese Academy of Medical Sciences and Peking Union Medical College, 100021 Beijing, China; 4 Department of General Surgery, Tongren Hospital, Shanghai Jiao Tong University School of Medicine (SJTU-SM), 200336 Shanghai, China; 5 Department of Orthopedic, the Affiliated Hospital of Jinazhou Medical University, 121001 Jinzhou, China; 6 Department of Occupational and Environmental Health, School of Public Health, Dalian Medical University, 116044 Dalian, China

**Keywords:** Colorectal cancer, Metastasis

## Abstract

Liver metastasis is the leading cause of death in colorectal carcinoma (CRC). However, little is known about the mechanisms of transferring effector messages between the primary tumor and the site of metastasis. Exosomes provide a novel transfer message method, and exosomal circular RNAs (circRNAs) play critical regulatory roles in cancer biology. In this study, the results showed that the expression of circPABPC1 was aberrantly upregulated in CRC tissues and exosomes. Exosomal circPABPC1 was considered an oncogene by functional experimental analysis in vitro and in vivo. Mechanistically, circPABPC1 recruited KDM4C to the HMGA2 promoter, reduced its H3K9me3 modification and initiated the transcription process in the nucleus. Moreover, cytoplasmic circPABPC1 promoted CRC progression by protecting ADAM19 and BMP4 from miR-874-/miR-1292-mediated degradation. Our findings indicated that exosomal circPABPC1 is an essential regulator in CRC liver metastasis progression by promoting HMGA2 and BMP4/ADAM19 expression. CircPABPC1 is expected to be a novel biomarker and antimetastatic therapeutic target in CRC.

## Introduction

Colon cancer (CRC) is the third most frequent cancer worldwide [1]. Approximately 30–50% of patients present with distant metastases, and liver metastasis is the leading cause of death in CRC [2, 3]. For CRC patients with liver metastasis, surgery is less effective, and most metastatic cancers develop resistance to adjuvant therapy [4, 5]. Therefore, further exploration of physiopathological mechanisms and improvement of the treatment of liver metastases of CRC patients are urgently needed. Recently, increasing data have shown that exosomes (40–150 nm) are essential mediators in intercellular communication and enclose various types of bioactive cargos [6, 7]. Among these bioactive components, noncoding RNAs (ncRNAs) are enriched and stable in exosomes, and they have important regulatory functions in the progression of cancers [8].

Circular RNAs (circRNAs) are a subtype of ncRNAs formed by alternative splicing called “back-splicing”, and they have a covalently closed loop structure and are more stable than linear RNAs [9, 10]. Owing to their stability and association with tumor progression, circRNAs have been considered future diagnostic biomarkers in cancers [11]. Previous studies have proposed that circRNAs show tissue/development stage specificity and function as essential regulators in cancer development and metastasis [11–13]. Accumulating evidence has demonstrated crucial functions of circRNAs, including functioning as microRNA (miRNA) sponges to regulate the transcript profile. For example, ciRS-7 is involved in the regulation of several genes via miR-7 sponging in the brain [14], and circ102049 is an antimetastatic target by regulating miR-761/miR-192-3p expression in CRC patients [15]. New techniques, such as RNA pull-down and RNA immunoprecipitation (RIP), have identified another important function of circRNAs, i.e., regulators of RNA-binding proteins (RBPs). Previous reports have shown that circPTK2 plays critical roles in CRC growth and metastasis by binding to vimentin protein at Ser38, Ser55, and Ser82 [16]. Exosomal circRNAs have been identified by RNA sequencing analyses of hepatic MHCC-LM3 cell-derived exosomes [11]. One of the hypotheses for the function of circRNAs in exosomes is cell-to-cell communication, which is involved in tumor metastasis [17]. A previous study has identified 16115 exosomal circRNAs from CRC patient serum [18]. However, the function of exosomal circRNAs in CRC progression, especially in liver metastasis, is still undefined.

In the present study, RNA sequencing and qPCR analysis were used to identify circPABPC1 in CRC tumors and exosomes [19]. We showed that the expression of circPABPC1 was high in tumor and liver metastasis tissues as well as in serum exosomes of CRC patients and cells. In addition, exosomal circPABPC1 was identified as an important regulator of CRC liver metastases by functional experimental analysis in vitro and in vivo. We further detailed the mechanisms by which circPABPC1 recruited KDM4C to the HMGA2 promoter, reduced its H3K9me3 modification and initiated the transcription process in the nucleus. Moreover, cytoplasmic circPABPC1 promoted CRC progression by protecting ADAM19 and BMP4 from miR-874-/miR-1292-mediated degradation. These findings suggested that circPABPC1 is a critical metastasis regulator of CRC, indicating that it may serve as a potential biomarker and therapeutic target in clinical practice.

## Results

### Expression profile of circRNAs in human CRC tissues

**Fig. 1:**
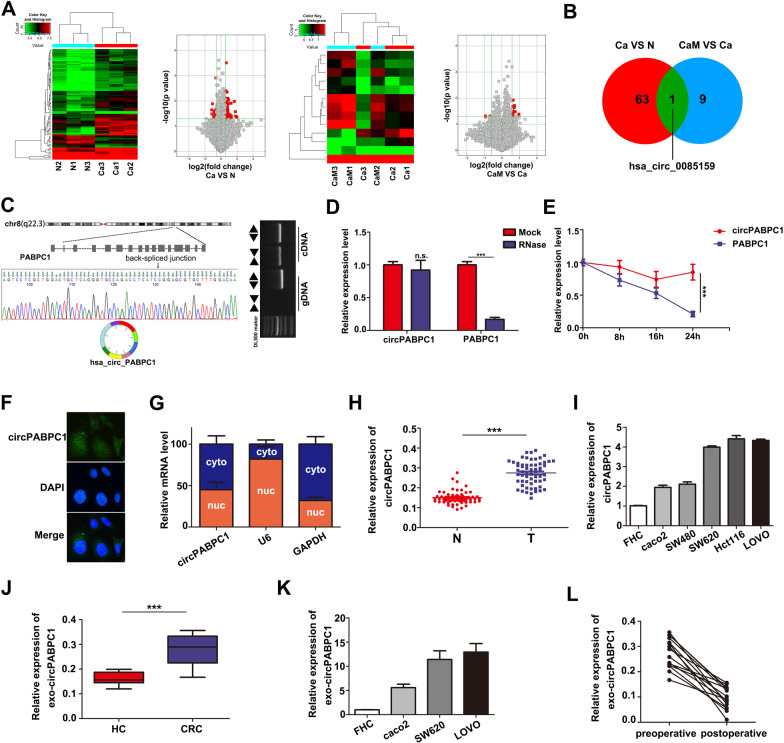
Identification of circPABPC1 as a biomarker for CRC. **A**, **B** Cluster heatmap and volcano map showing the differentially expressed circRNAs in paired human CRC tissues, distant metastasis tissues and normal tissues (*n* = 3). **C** Circularization of circPABPC1 was confirmed by qRT–PCR with divergent primers. **D** qRT–PCR analysis of circPABPC1 and PABPC1 mRNA after treatment with or without RNase R in CRC cells. **E** The expression of circPABPC1 and linear PABPC1 mRNA was detected by qRT–PCR after actinomycin D treatment. **F** The localization of circPABPC1 was measured by FISH assay. **G** Cytoplasmic and nuclear mRNA fractionation experiments were performed to detect the localization of circPABPC1. **H** The expression of circPABPC1 was measured by qRT–PCR in 60 pairs of CRC tissues and cells. N: normal tissues; T: CRC tissues. **I** The expression of circPABPC1 was measured by qRT–PCR in CRC cell lines. **J, K** The expression of circPABPC1 was measured by qRT–PCR in human blood sample exosomes and cell exosomes. **L** Levels of exosomal circPABPC1 in human blood samples before and after surgery. Quantitative data from three independent experiments are shown as the mean ± SD (error bars). *P < 0.05, ^**^P < 0.01, ^***^P < 0.001 (Student’s *t* test).

To identify differentially expressed circRNAs during CRC metastasis progression, we performed circRNA microarray assays and analyzed circRNA expression profiles from nine patient specimens (CaM, colorectal cancer with liver metastasis, *n* = 3; Ca, colorectal cancer without metastasis, *n* = 3; N, three adjacent normal tissues). As shown in Fig. 1A, a total of 64 upregulated circRNAs were identified (*P* < 0.05 and fold change > 1.5) in CRC tissues compared to normal tissues. In addition, 10 circRNAs were significantly upregulated in the CaM group compared to the Ca group. Among them, hsa-circ-0085159 was the only upregulated and conserved circRNA in the two different cohorts (Ca vs. N and CaM vs. Ca) (Fig. 1B). We then determined that hsa-circ-0085159 was spliced from exons 2 to 9 of the PABPC1 gene by using circBase and the UCSC database. The back-spliced junction of circPABPC1 was amplified and validated with divergent PCR primers (Fig. 1C). To further detect the essential properties of circPABPC1, both circPABPC1 and the PABPC1 linear mRNA were treated with RNase R and actinomycin D, a transcription inhibitor. The results showed that circPABPC1 was more resistant to both RNase R and actinomycin D, suggesting that circPABPC1 was more stable than linear PABPC1 (Fig. 1D, E). Furthermore, fluorescence in situ hybridization (FISH) using a biotin-labeled specific circPABPC1 probe and RNA fractionation were further performed to explore the localization of circPABPC1 (Fig. 1F, G). Surprisingly, a fluorescence signal was found in both the nucleus and cytoplasm. Moreover, the expression of PABPC1 was analyzed using (The Cancer Genome Atlas (TCGA) database (Fig. S1A). To clarify the correlation between PABPC1 gene amplification and circPABPC1 expression during CRC progression, we validated the expression pattern of circPABPC1 and PABPC1 mRNA in 60 pairs of clinical CRC tissues. The results showed that both circPABPC1 and PABPC1 were aberrantly upregulated in CRC tissues compared to normal tissues (Fig. 1H; Fig. S1B), and these results were further confirmed in CRC cells (Fig. 1I; Fig. S1C). Furthermore, as shown in fig. S1D, the expression of circPABPC1 was also upregulated in patients with distance metastasis (M1) compared with patients without distance metastasis (M0). To detect whether circPABPC1 is present in serum exosomes, we tested blood samples from 15 CRC patients and 15 healthy controls. As shown in Figs. S1E–G, exosomes derived from patient serum were identified by nanoparticle tracking analysis (NTA), western blot assays of protein biomarkers (TSG101 and CD63) and transmission electron microscopy (TEM). As expected, exosomal circPABPC1 derived from serum was more abundant in CRC patients than in healthy controls (Fig. 1J), and the same expression pattern was further validated in CRC cell-derived exosomes (Fig. 1K). The expression of exosomal circPABPC1 in M1 patients was also higher than in M0 patients (Fig. S1H). Moreover, the expression of exosomal circPABPC1 was significantly decreased in the same patients’ serum after the removal of tumors, indicating that CRC tissues are the origin of exosomal circPABPC1 (Fig. 1L). The above results demonstrated that circPABPC1 may be effectively delivered into the circulation system by exosomes, suggesting that circPABPC1 may have a prometastatic capability during CRC progression.

### Knockdown of circPABPC1 suppresses CRC cell proliferation, invasion and migration in vitro

**Fig. 2:**
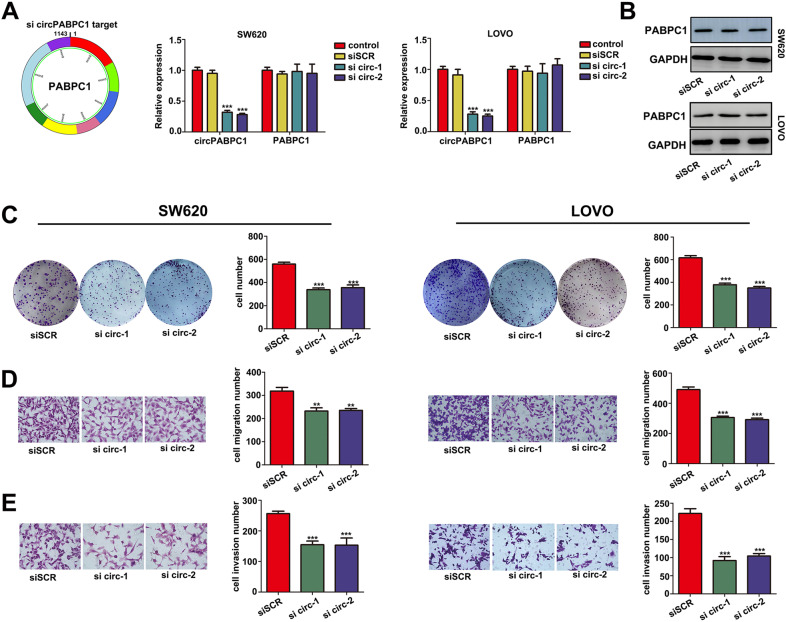
Knockdown of cirPABPC1 suppresses CRC cell proliferation and invasion in vitro. **A** CircPABPC1-specific siRNA significantly decreased circPABPC1 expression but had no effect on PABPC1 mRNA expression. **B** The protein level of PABPC1 was detected by western blot after si circPABPC1 transfection. **C**–**E** Colony formation, cell migration and invasion assays were used to detect the effect of circPABPC1 on cell proliferation and invasion. Quantitative data from three independent experiments are shown as the mean ± SD (error bars). ^*^P < 0.05, ^**^P < 0.01, ^***^P < 0.001 (Student’s *t* test).

Based on differential expression patterns in different CRC cells, we knocked down endogenous circPABPC1 in SW620 and LoVo cells using two siRNAs targeting the back-spliced sequence of circPABPC1, and these siRNAs had no effect on both PABPC1 linear mRNA and protein (Fig. 2A, B). To investigate the biological functions of circPABPC1 during CRC progression, multiple cell functional experiments were performed. Colony formation assays were used to assess the effect of circPABPC1 on CRC cell proliferation. As shown in Fig. 2C, knockdown of circPABPC1 significantly decreased the proliferation ability of both SW620 and LoVo cells, while circPABPC1 overexpression promoted the proliferation of SW480 cells (Fig. S2A). Matrigel invasion assays showed that the migration and invasion ability of SW620 cells were significantly downregulated after circPABPC1 knockdown (Fig. 2D, E). In contrast, functional experiments revealed that circPABPC1 overexpression significantly promoted migration and invasion in SW480 cells, indicating the essential role of circPABPC1 during CRC progression (Fig. S2B, C).

### CircPABPC1 regulates HMGA2 transcription in CRC cells via KDM4C and histone modification

**Fig. 3:**
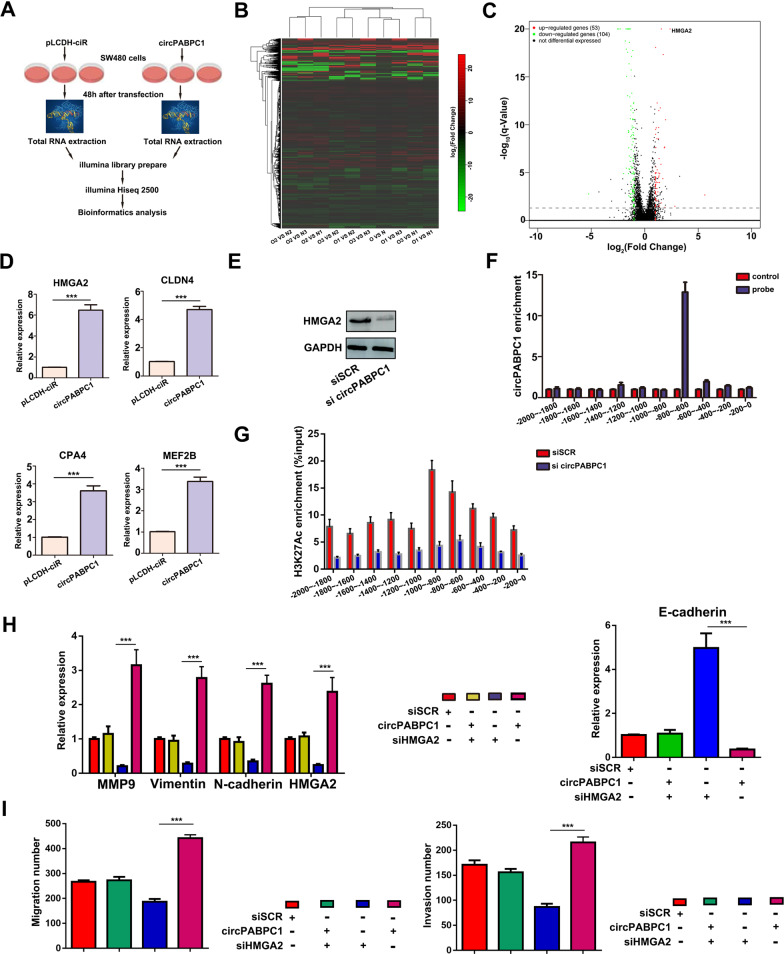
CircPABPC1 increases HMGA2 expression and regulates EMT progression in CRC. **A** Schematic illustration showing the details of circPABPC1 overexpression and RNA-seq. **B**, **C** Cluster heatmap and volcano map showing the differentially expressed genes after circPABPC1 overexpression in CRC cells. **D** The expression of four potential target genes was validated by qRT–PCR after circPABPC1 overexpression. **E** The protein level of HMGA2 was detected by western blot assay after circPABPC1 knockdown. **F** The enrichment of circPABPC1 on the promoter of HMGA2 was detected by CHIRP using circPABPC1-specific biotin-labeled probes. **G** The enrichment of H3K27Ac modification on the promoter of HMGA2 was detected by ChIP assay in CRC cells after circPABPC1 knockdown. **H** The protein levels of EMT-related genes were detected by western blot analysis. **I** The effect of circPABPC1 and HMGA2 on cell invasion was measured by cell migration and invasion assays. Quantitative data from three independent experiments are shown as the mean ± SD (error bars). ^*^P < 0.05, ^**^P < 0.01, ^***^P < 0.001 (Student’s *t* test).

To accurately clarify the specific molecular mechanism of circPABPC1 in CRC, next-generation sequencing was performed to identify the differentially expressed genes after circPABPC1 overexpression (Fig. 3A). As shown in Fig. 3B, C, 53 genes were upregulated and 104 genes were downregulated after circPABPC1 overexpression in CRC cells. Several significantly overrepresented molecular functions, including pathways involved in transcription factor activity, were identified by Gene Ontology (GO) and Kyoto Encyclopedia of Genes and Genomes (KEGG) enrichment analyses, suggesting that circPABPC1 may regulate the gene transcription process (Fig. S3A, B). Furthermore, Gene Set Enrichment Analysis (GSEA) of RNA-seq data demonstrated that circPABPC1 overexpression significantly regulated cell apoptosis progression and the JAK-STAT pathway (Fig. S3C, D). To confirm the relationship between circPABPC1 and cell apoptosis-related genes, qRT–PCR was performed after circPABPC1 overexpression. The results showed that the expression of proapoptotic genes, including caspase 9, caspase 8, Bad and Bax, was decreased after circPABPC1 overexpression (Fig. S3E). Among the multiple upregulated genes, high mobility group AT-hook 2 (HMGA2) was the most upregulated gene, which was further validated by qRT–PCR (Fig. 3D). Moreover, western blot assays confirmed that the protein level of HMGA2 was decreased after circPABPC1 knockdown (Fig. 3E). To validate whether circPABPC1 participates in the HMGA2 transcription process, chromatin isolation by RNA purification (CHIRP) assays with a specific biotin-labeled circPABPC1 probe was performed. The results showed that circPABPC1 was significantly enriched in the region of −800 to −600 bp from the transcription start site (TSS) of the HMGA2 promoter (Fig. 3F). In addition, circPABPC1 knockdown reduced the enrichment of histone H3K27Ac modifications on the HMGA2 promoter (Fig. 3G). The above results suggested that circPABPC1 may regulate HMGA2 expression at the transcriptional level. Because HMGA2 plays a pivotal role in cancer metastasis progression by regulating epithelial–mesenchymal transition (EMT) in various cancers, we detected the expression of EMT target genes, such as N-cadherin, E-cadherin and vimentin by western blot analysis (Fig. 3H). The results showed that the protein levels of vimentin, MMP9 and N-cadherin were decreased after inhibiting HMGA2 expression. The influence of siHMGA2 was reversed by upregulation of circPABPC1 in SW620 cells. Furthermore, the influence of siHMGA2 on cell migration and invasion was also reversed by circPABPC1 in CRC cells (Fig. 3I).

**Fig. 4:**
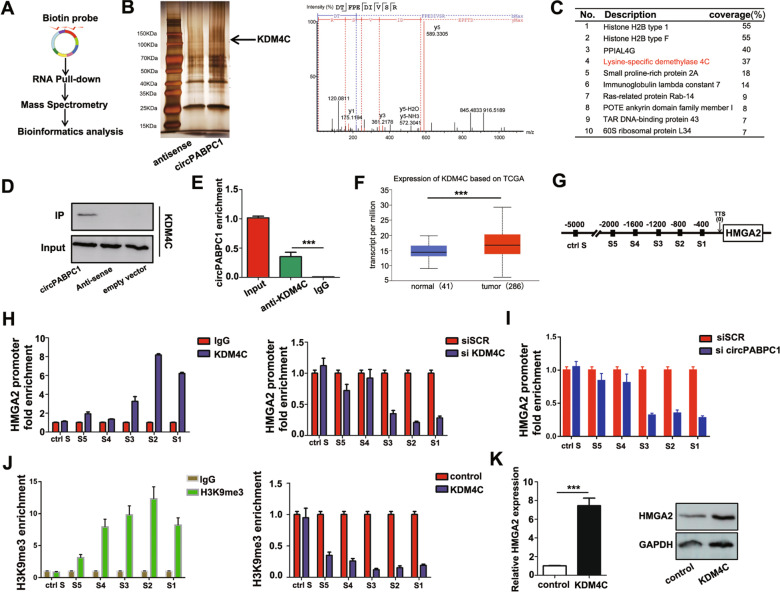
CircPABPC1 interacts with KDM4C and regulates H3K9me3 modification on the HMGA2 promoter. **A** Schematic illustration showing the details of circPABPC1 RNA pull-down and mass spectrometry. **B** Silver staining was used to illustrate the circPABPC1-precipitated proteins. **C** The top 10 precipitated proteins with high scores are shown. **D** The mass spectrometry results were further validated by western blotting. **E** The interaction of circPABPC1 and KDM4C was confirmed by RIP assay. **F** The expression of KDM4C in CRC patients was explored by TCGA database. **G**, **H** The enrichment of KDM4C on the promoter of HMGA2 was detected by ChIP assay. IgG was used as negative control. **I** The enrichment of KDM4C on the promoter of HMGA2 was detected by ChIP assay after circPABPC1 knockdown. **J** The enrichment of H3K9me3 modification on the HMGA2 promoter was detected by ChIP assay. IgG was used as negative control. **K** The effect of KDM4C on HMGA2 expression was measured by western blot analysis. Quantitative data from three independent experiments are shown as the mean ± SD (error bars). ^*^P < 0.05, ^**^P < 0.01, ^***^P < 0.001 (Student’s *t* test).

To further explore the specific mechanism of circPABPC1 in the HMGA2 transcription process, RNA pull-down assays followed by silver staining and mass spectrum identification were performed (Fig. 4A, B). As Fig. 4C shows, circPABPC1 cooperated with multiple nuclear proteins, including lysine-specific demethylase 4 C (KDM4C). The interaction between circPABPC1 and KDM4C was also confirmed by biotin-labeled circPABPC1 pull-down and RNA-protein immunoprecipitation (RIP) assays (Fig. 4D, E). By using TCGA pan-cancer analysis, we found that the expression of KDM4C was significantly higher in CRC than in normal tissues (Fig. 4F and S4A). We next analyzed the expression of KDM4C in CRC tissues and normal tissues. qRT–PCR analysis confirmed TCGA results and further demonstrated that patients with metastasis had higher KDM4C expression, suggesting that KDM4C may participate in tumor metastasis progression (Fig. S4B). We also found that CRC patients with higher KDM4C expression showed a poorer prognosis by Kaplan–Meier survival analysis based on TCGA database (Fig. S4C). To further determine whether KDM4C participates in the transcription process of HMGA2, chromatin immunoprecipitation (ChIP) assays were performed in CRC cells. As shown in Fig. 4G, H, KDM4C was enriched in the same region of the HMGA2 promoter as circPABPC1. The enrichment of KDM4C was also decreased after circPABPC1 knockdown (Fig. 4I). We also detected histone H3K9me3 modification levels in the HMGA2 promoter after KDM4C overexpression. The results showed that KDM4C significantly reduced the H3K9me3 modification of the HMGA2 promoter, thereby activating the transcription process (Fig. 4J). Moreover, our results also demonstrated that KDM4C increased HMGA2 expression at both the mRNA and protein levels (Fig. 4K). Taken together, these findings demonstrated that circPABPC1 is essential for KDM4C-mediated HMGA2 expression as circPABPC1 recruits KDM4C to the HMGA2 promoter, reduces its H3K9me3 modification and initiates the transcription process.

### CircPABPC1 serves as a sponge of miR-874 and miR-1292

**Fig. 5:**
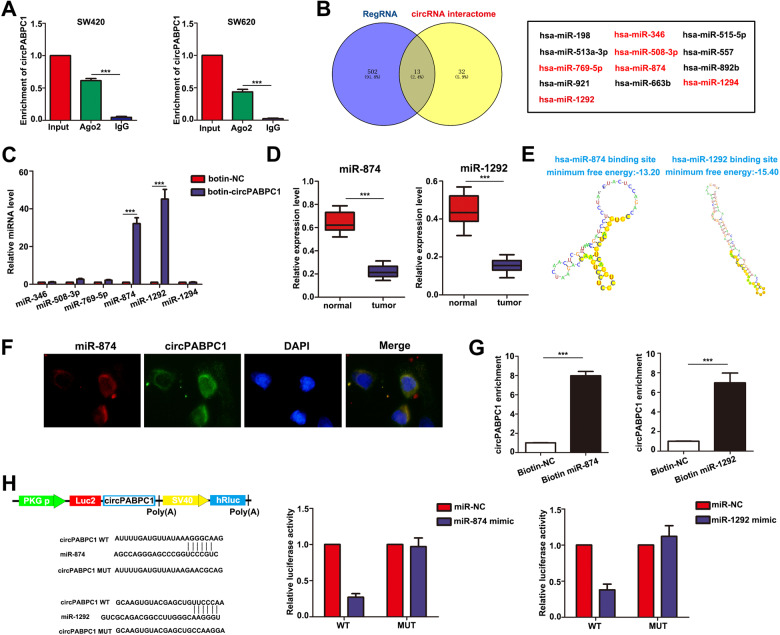
CircPABPC1 functions as a sponge for miR-874 and miR-1292 during CRC progression. **A** The interaction between circPABPC1 and AGO2 protein was confirmed by RIP assay in CRC cells. **B** The potential miRNAs that bind with circPABPC1 were predicted by the RegRNA and circRNA interactome databases. **C** CircPABPC1-specific probes were used to perform RNA RIP experiments. **D** The expression of miR-874 and miR-1292 was detected in CRC and adjacent normal tissues. **E** The secondary structure of potential miR-874-/miR-1292-binding sites in circPABPC1 was predicted by RegRNA. **F** The colocalization of miR-874 and circPABPC1 was confirmed by FISH assays. **G** A biotinylated RNA pull-down assay was performed to detect the miR-874- or miR-1292-captured circPABPC1 fractions. **H** Luciferase reporter activity was measured in CRC cells after cotransfection with circPABPC1-WT or circPABPC1-Mut and miR-874/miR-1292 mimic. Scale bar = 50 μm. Quantitative data from three independent experiments are shown as the mean ± SD (error bars). ^*^P < 0.05, ^**^P < 0.01, ^***^P < 0.001 (Student’s *t* test).

Given that circRNAs have been widely explored as miRNA sponges and circPABPC1 has been demonstrated to be present in the cytoplasm, we next investigated the specific miRNA interactions with circPABPC1. First, we confirmed the interaction between circPABPC1 and AGO2 using RIP assays in both SW480 and SW620 cells (Fig. 5A). Through overlapping the results of miRNA target prediction by RegRNA and the Circular RNA Interactome database, 13 candidate miRNAs were identified as putative targets of circPABPC1 (Fig. 5B). We then analyzed the expression of the above candidate miRNAs in CRC using the starBase database and found that only miR-874, miR-508 and miR-1292 were decreased in CRC tissues compared to normal tissues (Fig. S5). Furthermore, RNA pull-down assays demonstrated an enrichment of miR-874 and miR-1292 binding to circPABPC1, while the other miRNAs demonstrated no enrichment in CRC cells (Fig. 5C). Consistent with TCGA database, qRT–PCR analysis demonstrated that both miR-874 and miR-1292 were decreased in CRC (Fig. 5D). To confirm the biological functions of miR-874 and miR-1292 during CRC progression, we transfected miR-874 mimics or miR-1292 mimics into SW620 cells. Colony formation and Transwell assays showed that the miR-874 and miR-1292 mimics suppressed CRC cell proliferation, migration and invasion, suggesting that miR-874 and miR-1292 may function as tumor suppressors in CRC (Fig. S6A–C). To further confirm the interaction between circPABPC1 and miR-874/miR-1292, the specific binding sites of circPABPC1 and miR-874/miR-1292 were predicted using an in silico analysis of regulatory RNA elements (Fig. 5E). FISH assays demonstrated that circPABPC1 and miR-874 colocalized in the cytoplasm (Fig. 5F), indicating their binding. A biotin-labeled RNA pull-down assay also indicated absorption of circPABPC1 and miR-874/miR-1292 (Fig. 5G). Finally, by constructing wild-type or mutant circPABPC1 fragments containing the predicted binding site of the identified miRNA and inserting them downstream of the dual-luciferase reporter gene, we observed that miR-874 and miR-1292 mimics decreased the relative luciferase activity compared to the miR-NC mimics, while the luciferase activity of the circPABPC1 mutant group did not change (Fig. 5H). Taken together, these results suggested that circPABPC1 functions as a sponge of both miR-874 and miR-1292.

### ADAM19 and BMP4 are upregulated in CRC and are regulated by circPABPC1

**Fig. 6:**
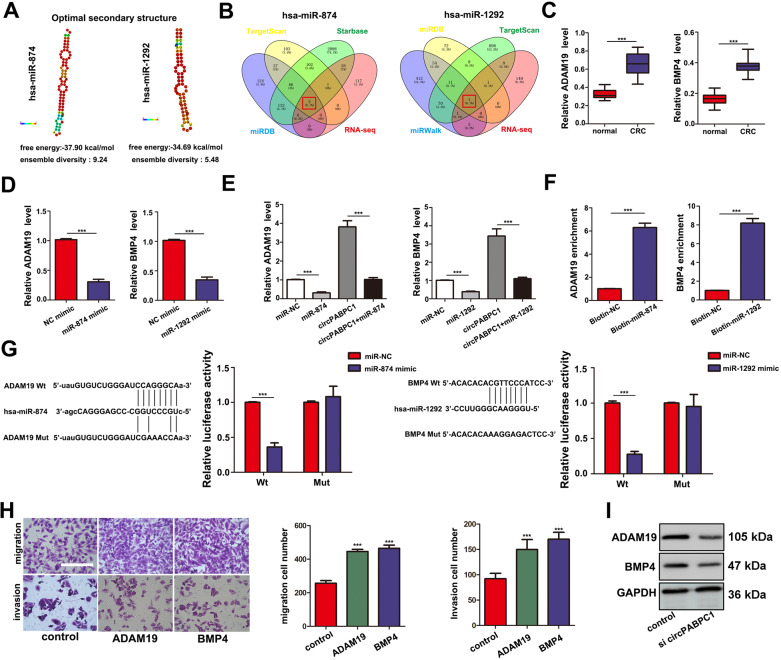
CircPABPC1 serves as a sponge for miR-874 and miR-1292 to increase ADAM19 and BMP4 in CRC cells. **A** The secondary structure and sequence conservation of miR-874 and miR-1292 was predicted by the optimal miRNA database. **B** Venn diagram showing the mutual putative target genes of miR-874 and miR-1292. **C** The expression of ADAM19 and BMP4 was detected in CRC tissues and adjacent tissues by qRT–PCR. **D** The expression of ADAM19 and BMP4 was measured by qRT–PCR after transfection of miR-874/miR-1292 mimics. **E** The expression of ADAM19 and BMP4 was measured by qRT–PCR in CRC cells after cotransfection with miR-874/miR-1292 mimics and circPABPC1. **F** Biotinylated RNA pull-down assay showing that the miR-874- or miR-1292-captured fractions specifically enrich ADAM19 and BMP4. **G** Luciferase reporter activity was measured in CRC cells after cotransfection with ADAM19/BMP4-WT or ADAM19/BMP4-Mut and miR-874/miR-1292 mimics. **H** The effect of ADAM19 and BMP4 on cell migration and invasion was measured by cell migration and invasion assays. **I** The expression of ADAM19 and BMP4 was measured by western blot analysis after circPABPC1 knockdown. Scale bar = 100 μm. Quantitative data from three independent experiments are shown as the mean ± SD (error bars). ^*^P < 0.05, ^**^P < 0.01, ^***^P < 0.001 (Student’s *t* test).

RNA fold software prediction allowed visualization of the mature and stable secondary structures of both miR-874 and miR-1292 (Fig. 6A). Through overlapping the results of miRNA target prediction by miRWalk, miRDB, TargetScan and our RNA-seq data, ADAM19 was identified as a putative target of miR-874, and BMP4 was identified as a putative target of miR-1292 (Fig. 6B, Fig. S7A). Through TCGA database analysis, we found that BMP4 expression was significantly higher in CRC tissues than in normal tissues and other types of tumors (Fig. S7B, C). The expression of ADAM19 was also aberrantly higher in CRC and associated with nodal metastasis (Fig. S7D, E). We also confirmed the expression of ADAM19 and BMP4 in CRC patients by qRT–PCR analysis (Fig. 6C). The mRNA levels of ADAM19 and BMP4 were significantly inhibited after miR-874/miR-1292 overexpression (Fig. 6D), while circPABPC1 cotransfection reversed this inhibition (Fig. 6E), indicating that circPABPC1 sponges miR-874/miR-1292 and modulates ADAM19 and BMP4 expression. In addition, biotin RNA pull-down also demonstrated that miR-874/miR-1292 directly interacted with ADAM19/BMP4 (Fig. 6F). The dual-luciferase assay demonstrated that miR-874 mimics led to decreased fluorescence of wild-type ADAM19 and that miR-1292 mimics significantly decreased the luciferase reporter activity of wild-type BMP4 (Fig. 6G). Moreover, ADAM19 and BMP4 overexpression also significantly enhanced the migration and invasion of CRC cells (Fig. 6H). CircPABPC1 knockdown also decreased the protein levels of ADAM19 and BMP4 (Fig. 6I). Taken together, these findings demonstrated that cytoplasmic circPABPC1 promotes CRC progression by protecting ADAM19 and BMP4 from miR-874-/miR-1292-mediated degradation.

### CircPABPC1 knockdown attenuates CRC progression and decreases HMGA2, BMP4 and ADAM19 expression in vivo

**Fig. 7:**
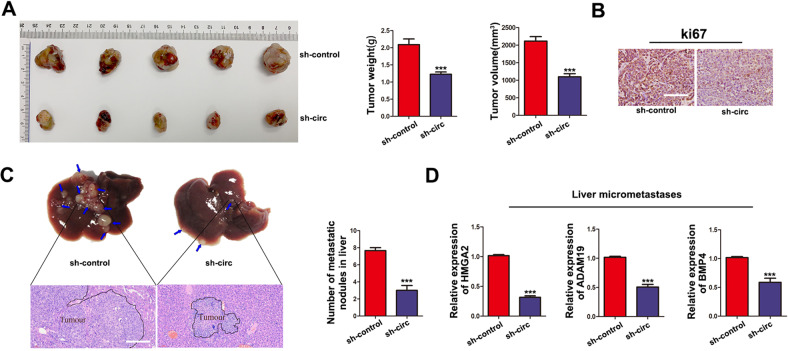
Knockdown of circPABPC1 suppresses CRC growth and liver metastasis in vivo. **A** Subcutaneous tumor formation was used to detect the effect of circPABPC1 on cell proliferation ability in vivo. **B** Ki-67 protein levels were measured by immunohistochemistry analysis in subcutaneous tumor tissues. **C** A liver metastasis model was used to measure cell invasion in vivo. **D** The expression of several genes was detected by qRT–PCR in liver micrometastasis. Scale bar = 200 μm. Quantitative data from three independent experiments are shown as the mean ± SD (error bars). ^*^P < 0.05, ^**^P < 0.01, ^***^P < 0.001 (Student’s *t* test).

A subcutaneous tumor formation model was used to detect the function of circPABPC1 in vivo. As shown in Fig. 7A, the growth of tumors from the sh-circPABPC1 group was significantly decreased compared to that of the NC group. Moreover, circPABPC1 knockdown also decreased cell proliferation in vivo (Fig. 7B). To determine the effect of circPABPC1 on the metastasis of CRC cells in vivo, a spleen injection experiment was performed. Compared to the NC group, lower liver metastases were observed in the sh-circPABPC1 group (Fig. 7C). In addition, the mRNA levels of HMGA2, ADAM19 and BMP4 were detected in liver metastases of mice by qRT–PCR analysis. Compared to the NC group, the expression of HMGA2, ADAM19 and BMP4 was decreased in the sh-circPABPC1 group (Fig. 7D). Moreover, to estimate the impact of Exo-circPABPC1 on CRC tumor formation in vivo. SW480 cells were injected subcutaneously into nude mice to form tumor masses. One week later, Exo-circPABPC1 or Exo-Vector was subsequently injected into the mice every 3 days. As shown in fig. S8, both volume and weight of tumors were higher in Exo-circPABPC1 groups compared with control. The above results suggested that circPABPC1 promotes CRC cell growth and metastasis in vivo.

## Discussion

The liver is the most common metastatic site of colorectal cancer (CRC), and 30% to 50% of CRC patients have hepatic metastasis during the development of the disease [20]. The primary curative treatment for CRC patients with liver metastases is resection. However, surgical resection or chemotherapy is unsatisfactory for these patients’ treatment due to rapid CRC metastasis deterioration and drug resistance [21, 22]. Therefore, liver metastasis is the leading cause of cancer-related mortality in CRC. To significantly improve the overall survival rate of CRC patients with liver metastasis, recent research has focused on novel biological agents against metastatic CRC. Recent studies have verified that circRNAs play vital roles in the progression of tumor metastasis and that they can be transferred by exosomes [11]. Exosomal circRNAs are involved in crosstalk between tumor cells and the microenvironment, and they are novel biomarkers and antimetastatic therapeutic targets [23].

In the present study, circRNA microarray analysis identified an oncogenic circRNA, namely, circPABPC1, which was upregulated in CRC tissues compared to matched normal tissues. Moreover, qPCR analysis demonstrated that exosomal circPABPC1 derived from serum was more abundant in CRC patients. To explore the biological functions of circPABPC1, Transwell migration and invasion assays were used to demonstrate the ability of circPABPC1 to promote tumor metastasis in vitro, and a hepatic metastasis model was generated to verify the effect of circPABPC1 on the metastasis of CRC in vivo. Together, these findings suggested that circPABPC1 contributed to the metastasis progression in CRC and that serum exosomal-circPABPC1 may be a potential mediator of intercellular communication in CRC development.

It has been well demonstrated that many circRNAs are located in the cytoplasm and perform regulatory functions by miRNA sponging; circRNAs are rarely located in the nucleus to regulate translation [24]. Herein, FISH experiments showed that circPABPC1 was expressed in both the nucleus and cytoplasm, suggesting the important regulatory functions of circPABPC1 in CRC metastasis development by sponging miRNAs and mediating translation. Our present work showed that cytoplasmic circPABPC1 promotes CRC progression by protecting ADAM19 and BMP4 from miR-874-/miR-1292-mediated degradation. Previous studies have demonstrated that ADAM19 [25–28] and BMP4 [29–35] are important activators by interacting with the EMT process, in which epithelial cells lose their cell polarity and cell–cell adhesion as well as gain a motile mesenchymal cell phenotype that is thought to cause metastasis [36]. Moreover, Transwell assays showed that overexpression of ADAM19 or BMP4 significantly enhanced the migration and invasion of CRC cells. Our present work showed that circPABPC1 acts as an important oncogene by regulating the expression of ADAM19 and BMP4, which are required for EMT-related metastasis in CRC. Recently, a previous study has reported that circRNAs alter the histone modification pattern by recruiting KAT7 to gene promoter regions as a molecular scaffold, thereby increasing H4K5 acetylation in their promoter regions [37]. Based on these findings, we identified another function of circPABPC1, i.e., regulators of RBPs, which recruit KDM4C to the HMGA2 promoter, reduce H3K9me3 modification and initiate transcription processes in the nucleus. Several studies have supported that HMGA2 plays an oncogenic role in the processes of CRC metastasis and participates in many aspects of cellular processes, including EMT [38–40]. In the present study, western blot analysis showed that the protein levels of vimentin, MMP9 and N-cadherin were decreased after inhibiting HMGA2 expression, and this influence was reversed by upregulation of circPABPC1 in SW620 cells. This is the first study to identify a critical role for exosomal circPABPC1 in inducing EMT by regulating ADAM19, BMP4 and HMGA2 in the processes of CRC liver metastasis. However, the present study had several limitations. The small number of CRC blood samples containing exosomal circPABPC1 was a major limitation. In the future, we will focus on the effect of prognostic predictors of exosomal circPABPC1 using a large number of CRC tissue and blood samples.

## Conclusions

**Fig. 8:**
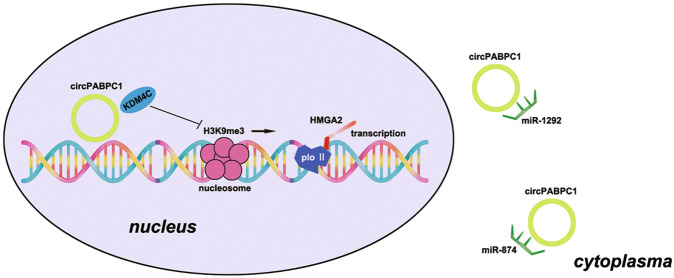
Mechanism for the regulatory function of circPABPC1 regulates HMGA2 and BMP4/ADAM19 in CRC progression.

In summary, as shown in Fig. 8A, our results indicated for the first time that exosomal circPABPC1 participates in the regulation of CRC liver metastasis. Additionally, we also demonstrated that circPABPC1 recruits KDM4C to the HMGA2 promoter, reduces its H3K9me3 modification and initiates transcription in the nucleus, and we also showed that cytoplasmic circPABPC1 promotes CRC progression by protecting ADAM19 and BMP4 from miR-874-/miR-1292-mediated degradation. This study provided a novel mechanism regarding the crosstalk between CRC liver metastasis and EMT mediated by exosomal circPABPC1, which is expected to be a novel biomarker and antimetastatic therapeutic target in CRC liver metastasis.

## Materials and methods

### Patient samples

A total of 60 paired CRC and adjacent normal tissues were obtained from the Surgery Department of First Affiliated Hospital of JinZhou Medical University. All fresh tissue specimens were immediately stored at −80 °C until RNA extraction. Twenty CRC patients and 20 normal control blood samples were collected for exosome extraction. This study was approved by the Ethics Committee of the First Affiliated Hospital of JinZhou Medical University.

### Total RNA extraction and qRT–PCR

In brief, total RNA was extracted from tissues or cells using TRIzol reagent (Life Technologies, Carlsbad, CA, USA). The concentration of total RNA was measured by Qubit 4.0. Then, 2 μg of isolated RNA was used for the reverse transcription reaction with the PrimeScript reverse transcriptase (RT) reagent kit (TaKaRa, Shiga, Japan). Finally, qRT–PCR was performed using SYBR green PCR mix (Takara). GAPDH or U6 served as internal controls.

### Cell lines, plasmids and transfection

All the cell lines used were obtained from American Type Culture Collection (ATCC, USA). For pLCDH-circPABPC1 plasmid generation, the sequences of exons 2-9 of PABPC1 were cloned into a pLCDH-ciR-vector (Invitrogen, Carlsbad, CA, USA). The pLCDH-ciR empty vector was used as a negative control. Cells were seeded in 6-well plates, and Lipofectamine 3000 (Invitrogen, Carlsbad, CA) was used according to the manufacturer’s instructions for transient transfections.

### RNA pull-down and mass spectrometry

circPABPC1-specific RNA pull-down was performed using a Magnetic RNA Protein Pull-down Kit (Thermo). A 5’ biotin-labeled oligonucleotide probe targeting the junction site of circPABPC1 was obtained from KeyGEN Company (Jiangsu, China). All sequences used are listed in Table S1. A total of 10^7^ CRC cells were transfected with the pLCDH-circPABPC1 overexpression plasmid or negative control. Forty-eight hours after transfection, total RNA was isolated and incubated with probes and streptavidin magnetic beads. Unbound proteins were washed away, and bound proteins were collected for silver staining and mass spectrometry.

### RIP assay

The RIP assay was performed using the MagnaRIP RNA-Binding Protein Immunoprecipitation Kit (Merck Millipore) according to the manufacturer’s instructions. In brief, cell lysates were incubated with magnetic beads coated with protein A/G. AGO2 (5 µg) and KDM4C primary antibody (Abcam, MA, USA) were added and incubated at 4 °C overnight. circPABPC1 and miRNAs were then detected by qRT–PCR.

### ChIP assay

Briefly, CRC cells were fixed with 4% paraformaldehyde and then incubated with glycine for 10 min. After glycine treatment, cells were lysed with ChIP lysis buffer. The whole genome DNA was then sonicated to 400~800 bp fragments. The lysates were immunoprecipitated with magnetic protein A/G beads conjugated with anti-H3K27Ac (rabbit monoclonal; Abcam) or rabbit nonimmune IgG (negative control). Finally, the precipitated DNA was analyzed by PCR.

### Western blot analysis

In brief, RIPA buffer was used to lyse cells, and total proteins were separated by 10% SDS–PAGE and electroblotted onto a PVDF membrane (Millipore). After blocking with nonfat milk for 1 h, membranes were incubated with specific primary antibodies overnight. The membranes were then washed with PBS three times and then incubated with horseradish peroxidase-coupled secondary antibodies (Abcam, MA, USA) for 2 h. After washing with PBS, the protein bands were detected using an ECL western blotting kit (Amersham Biosciences, UK).

**Table:** 

antibody	vendor	catalog number
PABPC1	abcepta	AP2920c
HMGA2	abcam	ab207301
MMP9	abcam	ab76003
Vimentin	abcam	ab92547
N-cadherin	abcepta	AP52149
E-cadherin	abcepta	AP1477a
KDM4C	abcepta	AP11444b
ADAM19	abcepta	AP9815c
BMP4	abcam	ab124715
Ki67	abcam	ab15580

### Exosome extraction and identification

Human serum exosomes were extracted from patient blood samples using ExoQuick Exosome Precipitation Solution (SBI, CA, USA) according to the manufacturer’s instructions. TEM and NTA were used to identify exosomes. The protein markers of exosomes, including TSG101, CD63 and CD81, were detected by western blot assay.

### Dual-luciferase reporter assay

In brief, HEK-293T cells were seeded in 96-well plates at a density of 1 × 10^4^ per well and cultured overnight. Cells were then cotransfected with a mixture of pmirGLO-wild circPABPC1 or pmirGLO-mutant circPABPC1 plasmids and miR-874/miR-1292 mimics using Lipofectamine 3000 (Invitrogen). Twenty-four hours later, the luciferase activity was detected by a dual-luciferase reporter assay system (Promega, WI, USA).

### Bioinformatics analysis and circRNA/miRNA/mRNA network construction

CRC-relevant RNA-Seq data from TCGA were downloaded from TCGA database (https://portal.gdc.cancer.gov). The candidate miRNAs were predicted by the RegRNA and circRNA interactome databases. The TargetScan, starBase, miRDB and miRWalk databases were used to detect miRNAs and their target genes. Cytoscape software was used to construct circRNA/miRNA/mRNA networks.

### Functional assay

The colony formation, Transwell migration and Matrigel invasion assays were performed according to our previously published study [41].

### Animal experiments

Xenograft assays were performed to detect the effect of circPABPC1 on the proliferation of CRC cells in vivo. In brief, 2 × 10^6^ SW620 cells in 0.1 mL of phosphate-buffered saline were subcutaneously injected into the flanks of 4-week-old male nude mice. One month later, the mice were sacrificed, and tumor tissues were collected.

To estimate the impact of Exo-circPABPC1 on CRC tumor formation in vivo, SW480 cells were subcutaneously injected into the right flank of nude mice. These mice were randomly separated into two groups (5 mice per group). One week later, the collected exosomes containing circPABPC1 or Vector was subsequently injected into mice via the tail vein (5 μg/mouse) every 3 days. One month later, the mice were sacrificed, and tumor tissues were collected.

A hepatic metastasis model was used to detect the effect of circPABPC1 on the metastasis of CRC cells in vivo. Mice were anesthetized, and 2 × 10^6^ SW620 cells were injected into the spleen with a 30-gauge needle. Six weeks later, the mice were sacrificed, and their livers were dissected out and further measured.

### Statistical analysis

SPSS 20.0 software and GraphPad Prism 5 were used to perform statistical analyses. Data are presented as the mean ± SD as indicated. Comparisons between two groups were assessed using an unpaired two-tailed *t* test. Statistical significance was expressed as a *P* value (^*^P < 0.05; ^**^P < 0.01; ^***^P < 0.001; and n.s., *P* > 0.05) as indicated in the individual figure legends.

## Supplementary information

SUPPLEMENTAL MATERIAL

Supplemental materials-Original blot

## Data Availability

The datasets generated during and/or analyzed during the current study are available from the corresponding author on reasonable request.
