# Supplementary material for: Exosomal circPABPC1 promotes colorectal cancer liver metastases by regulating HMGA2 in the nucleus and BMP4/ADAM19 in the cytoplasm
Source: Cell Death Discov. 2022 Jul 23;8:335. doi: 10.1038/s41420-022-01124-z (PMC9308786; doi:10.1038/s41420-022-01124-z)
Supplement: Supplementary file 1 — SUPPLEMENTAL MATERIAL [file 41420_2022_1124_MOESM1_ESM.docx]

**Table S1**

**Probe sequences of hsa_circPABPC1**

| **Name** | **Sequence** |
| --- | --- |
| hsa_circPABPC1_008-probe-biotin | AGACCTCATCCGGAGCGTGC |
| sa_circPABPC1_008-Negative-probe-biotin | GCACGCTCCGGATGAGGTCT |

**Figure S1**


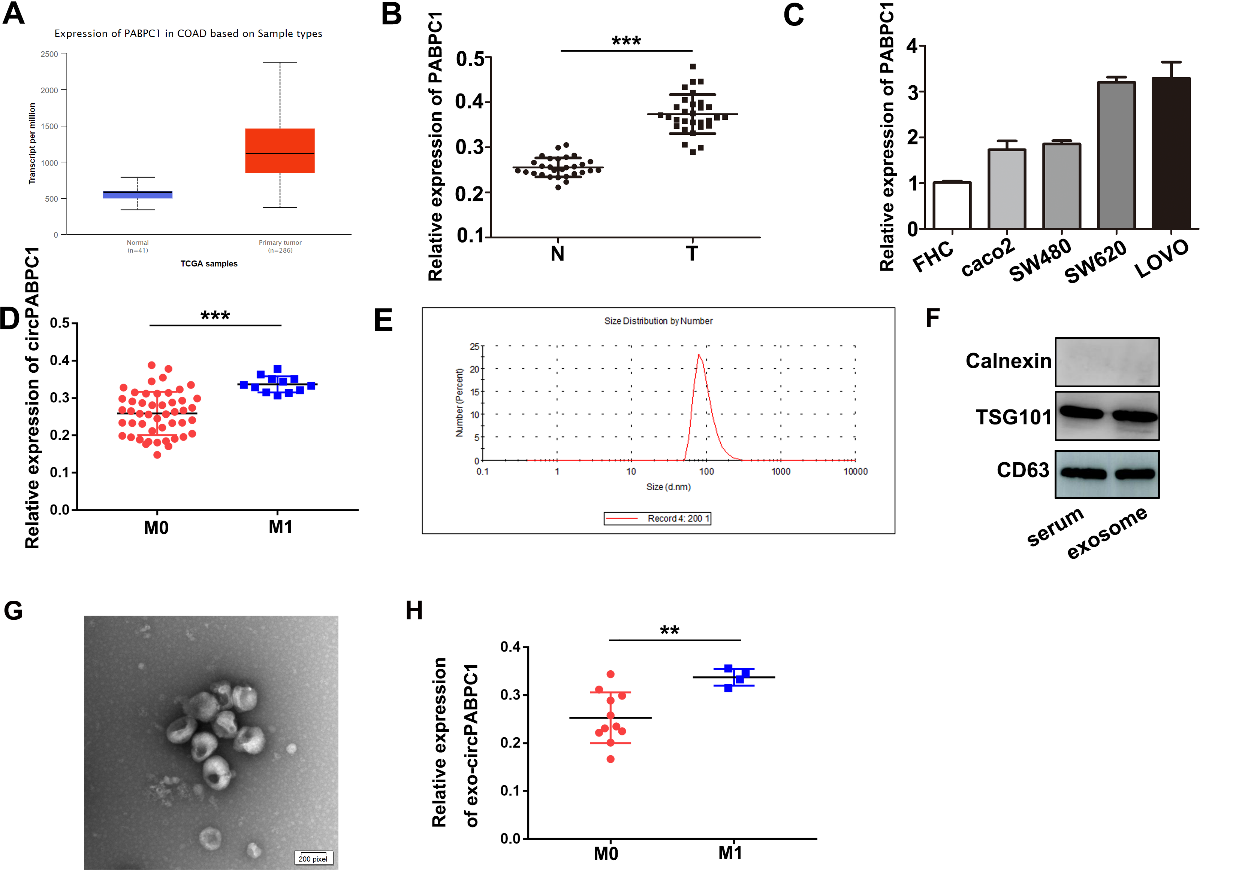


**Figure S1.** (A) The expression of PABPC1 in CRC and normal tissues was analysed by using TCGA database. (B) Relative PABPC1 expression level was measured by qRT-PCR in CRC tissues. N: normal; T: tumor. (C) PABPC1 expression pattern was detected in normal colonic epithelial cells and CRC cells. (D) The expression of circPABPC1 was measured by qRT-PCR in patients with distance metastasis (M1) and patients without distance metastasis (M0). (E-F) The presence of exosomes in human blood samples was identified by NAT and western blot. (G) The presence of exosomes in human blood samples was identified by TEM. (H) The expression of exosomal circPABPC1 was measured by qRT-PCR in patients with distance metastasis (M1) and patients without distance metastasis (M0).

**Figure S2**

**
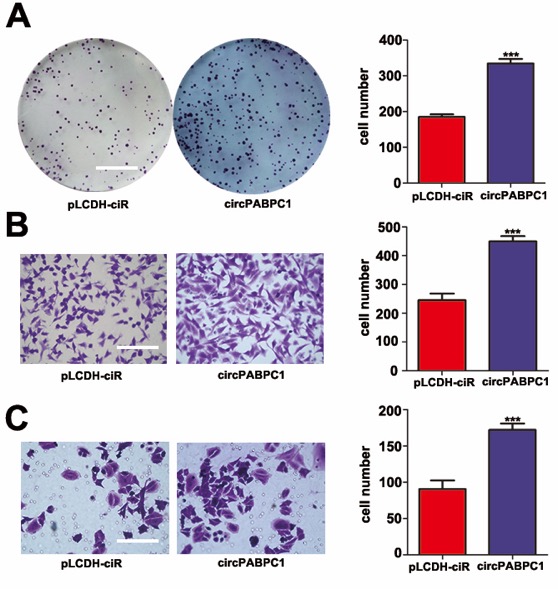
**

**Figure S2.** (A-C) The proliferation, migration and invasion of SW480 cells was measured by colony formation **A**, cell migration **B** and invasion **C** assays after circPABPC1 overexpression. Scan bar=100 μm.

**Figure S3**

**
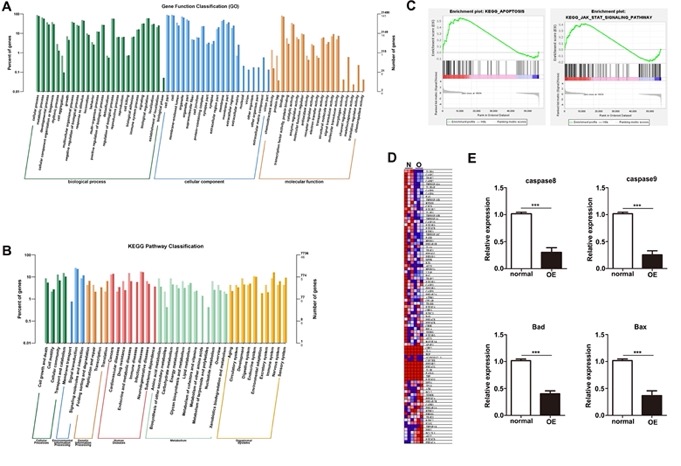
**

**Figure S3.** (A-C) RNA-seq analysis was performed in CRC cells after circPABPC1 overexpression. The datasets were analysed by GO **A**, KEGG **B** and GSEA **C**, using the Hallmark gene signature collection. (D) GSEA analysis indicated a significant correlation between circPABPC1 expression and cell apoptosis-related gene signatures. (E) The expression of apoptosis-related genes was measured by qRT-PCR after circPABPC1 overexpression. OE: overexpression.

**Figure S4**

**
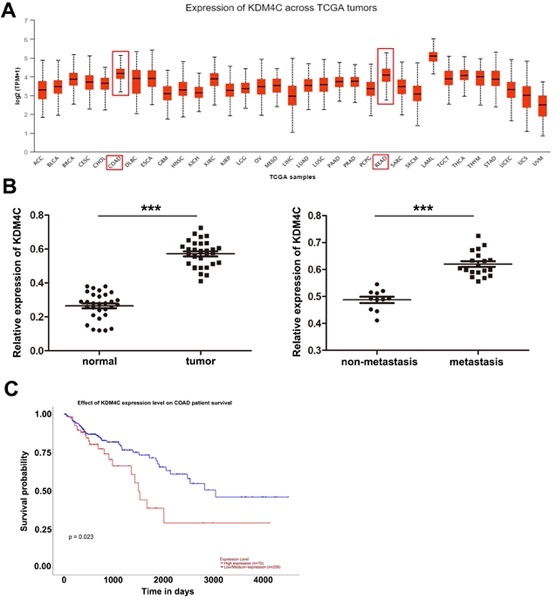
**

**Figure S4.** (A) The expression of KDM4C across tumors was analysed by TCGA Pan-cancer. (B) KDM4C expression level in CRC tissues were measured by qRT-PCR. (C) Kaplan-Meier’s survival curve indicated the high KDM4C expression is correlated with lower CRC survival rates by TCGA database.

**Figure S5**

**
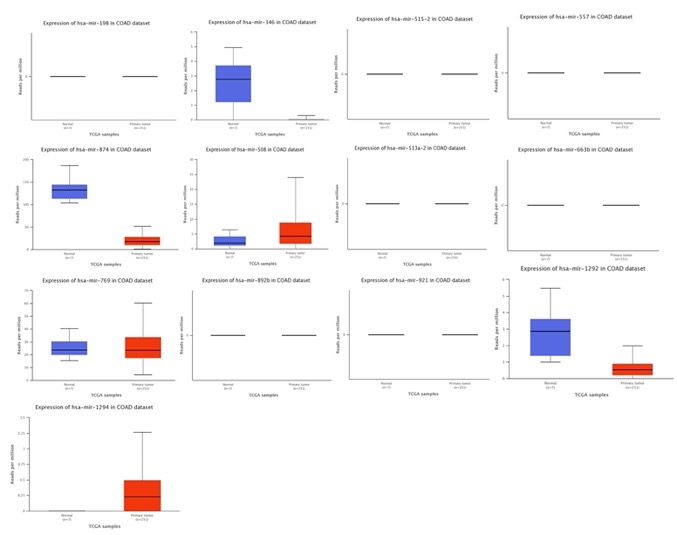
**

**Figure S5.** Analysis of miRNAs expression in CRC by Starbase 3.0 database.

**Figure S6**

**
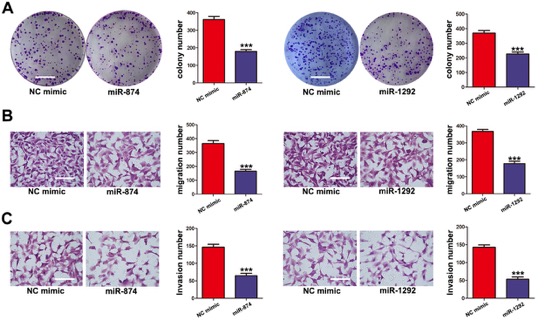
**

**Figure S6.** (A-C) Colony formation **A**, cell migration **B** and Transwell **C** assays showed that both miR-874 and miR-1292 decreased the proliferation and metastasis of CRC cells. Scan bar=100 μm.

**Figure S7**

**
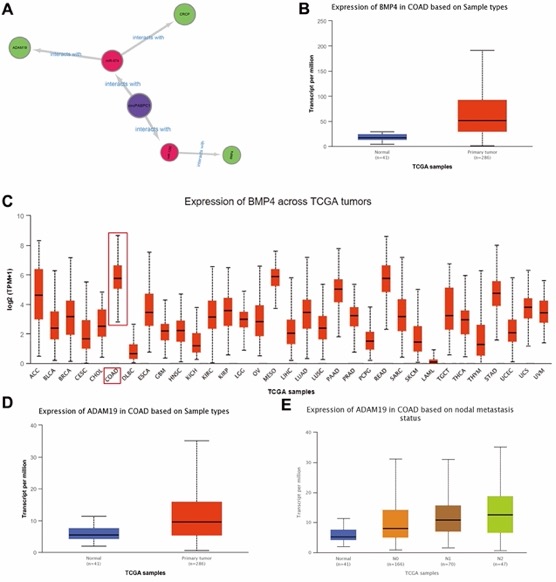
**

**Figure S7.** (A) The circRNA/miRNA/mRNA network was established by cystoscope software. (B) Expression of BMP4 in TCGA samples was shown. (C) The expression of BMP4 across tumors was analysed by TCGA Pan-cancer. (D-E) Expression of ADAM19 in TCGA samples was shown.

**Figure S8**

**
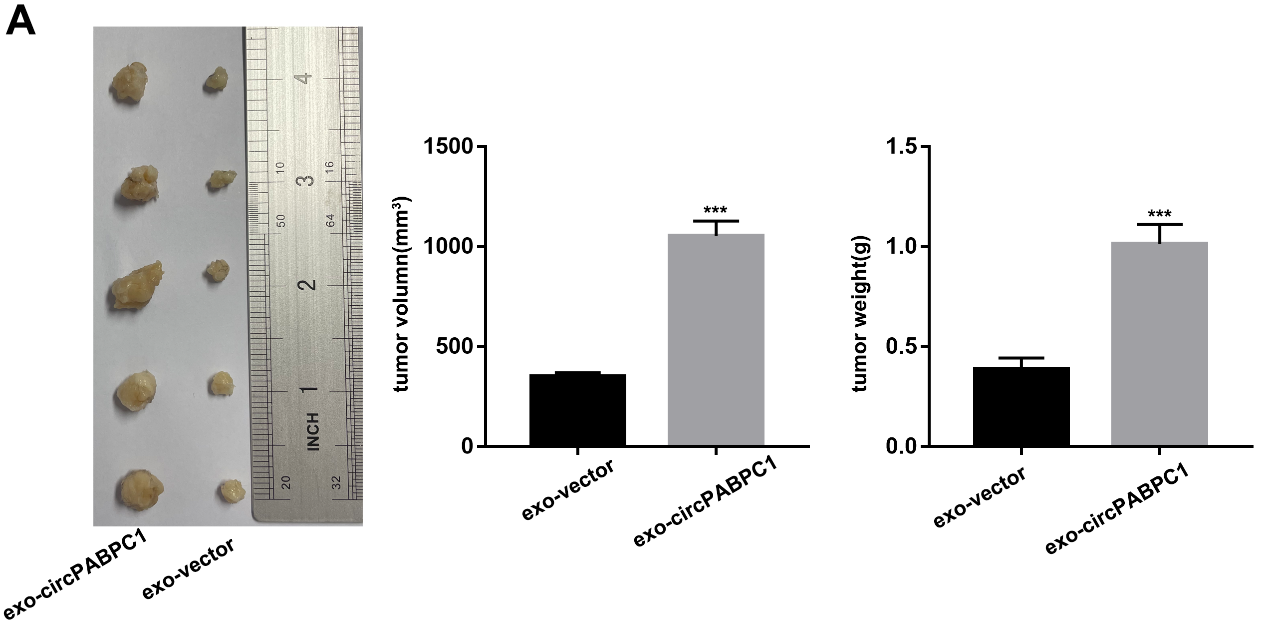
**

**Figure S8.** Exosomal circPABPC1 promoted tumor growth in vivo. SW480 cells were inoculated subcutaneously into the nude mice. 1 week later, Exo-circPABPC1 or Exo-

Vector was subsequently injected into mice. Tumor volume and weight were measured after one month later.
