# Supplementary material for: Exosomal circPABPC1 promotes colorectal cancer liver metastases by regulating HMGA2 in the nucleus and BMP4/ADAM19 in the cytoplasm
Source: Cell Death Discov. 2022 Jul 23;8:335. doi: 10.1038/s41420-022-01124-z (PMC9308786; doi:10.1038/s41420-022-01124-z)

**Figure 2B**

PABPC1-SW620


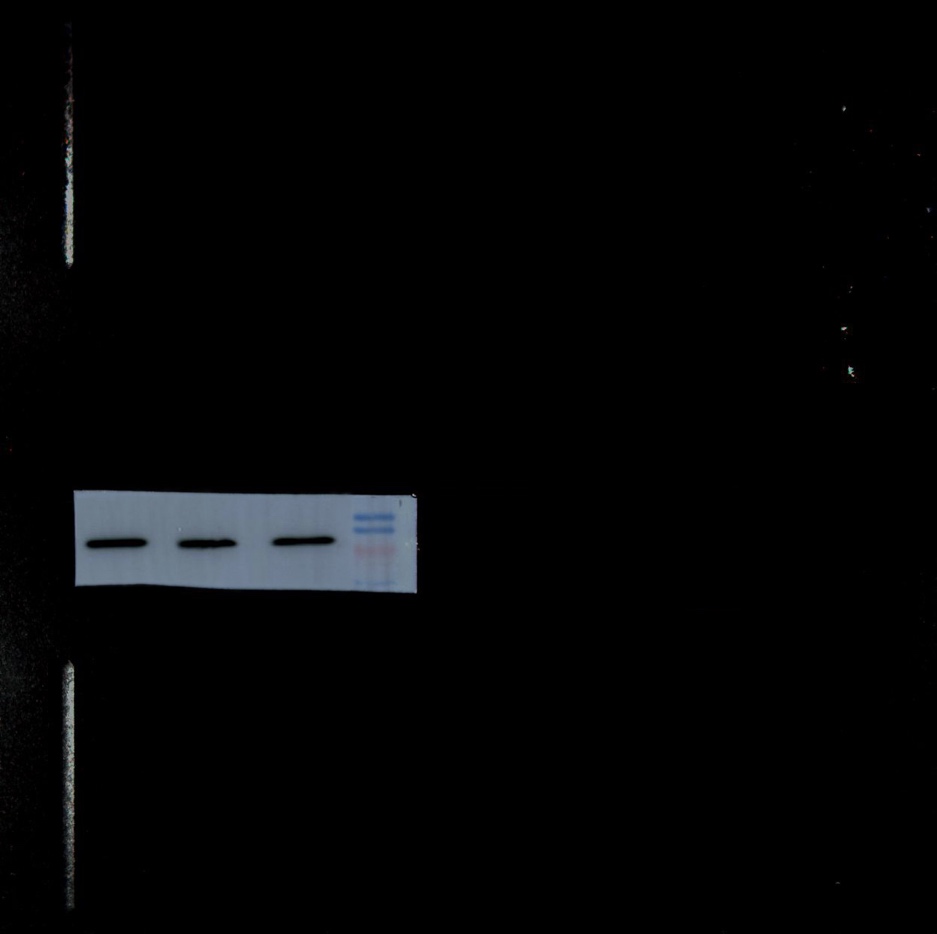


GAPDH-SW620


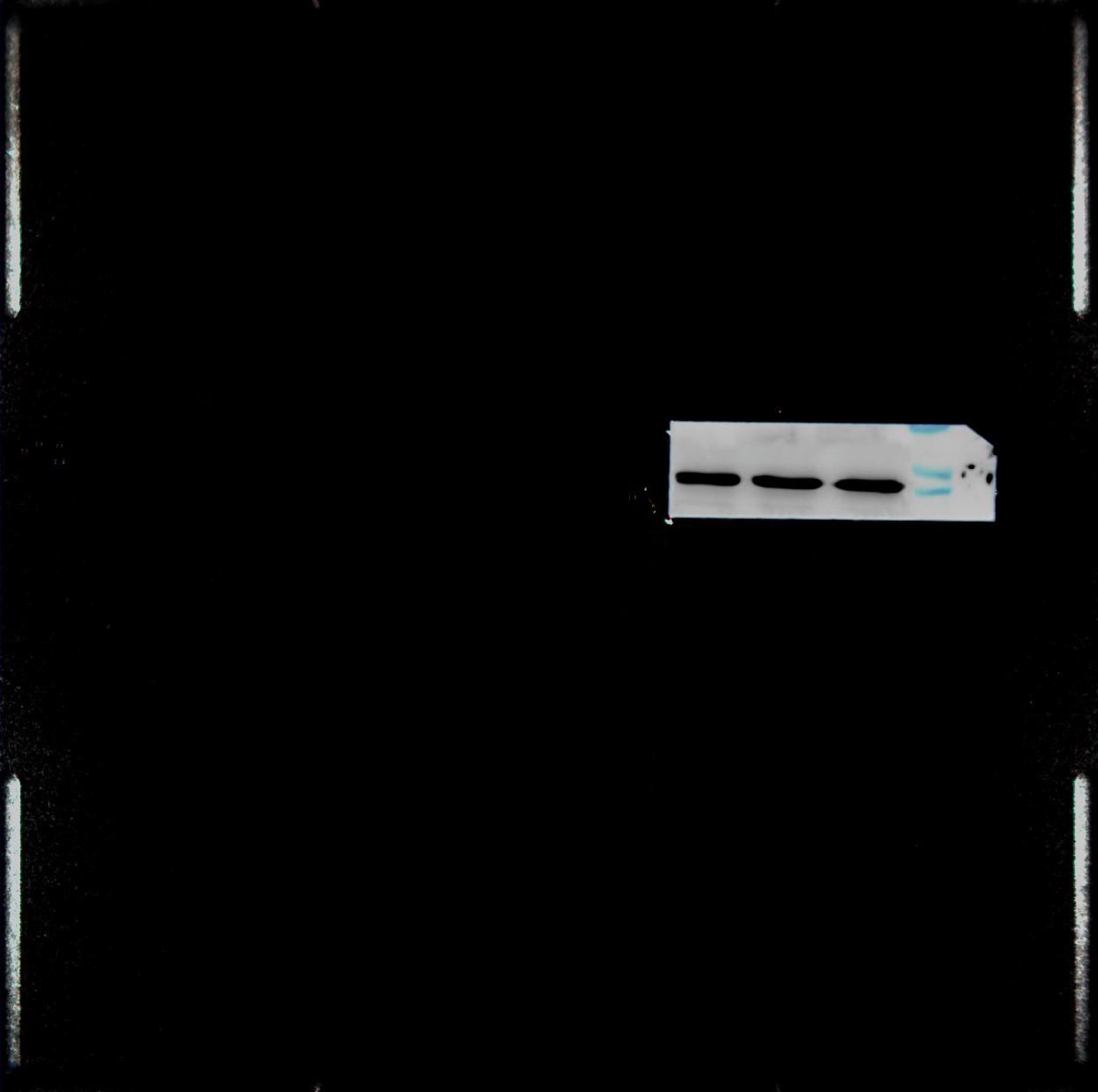


PABPC1-LoVo


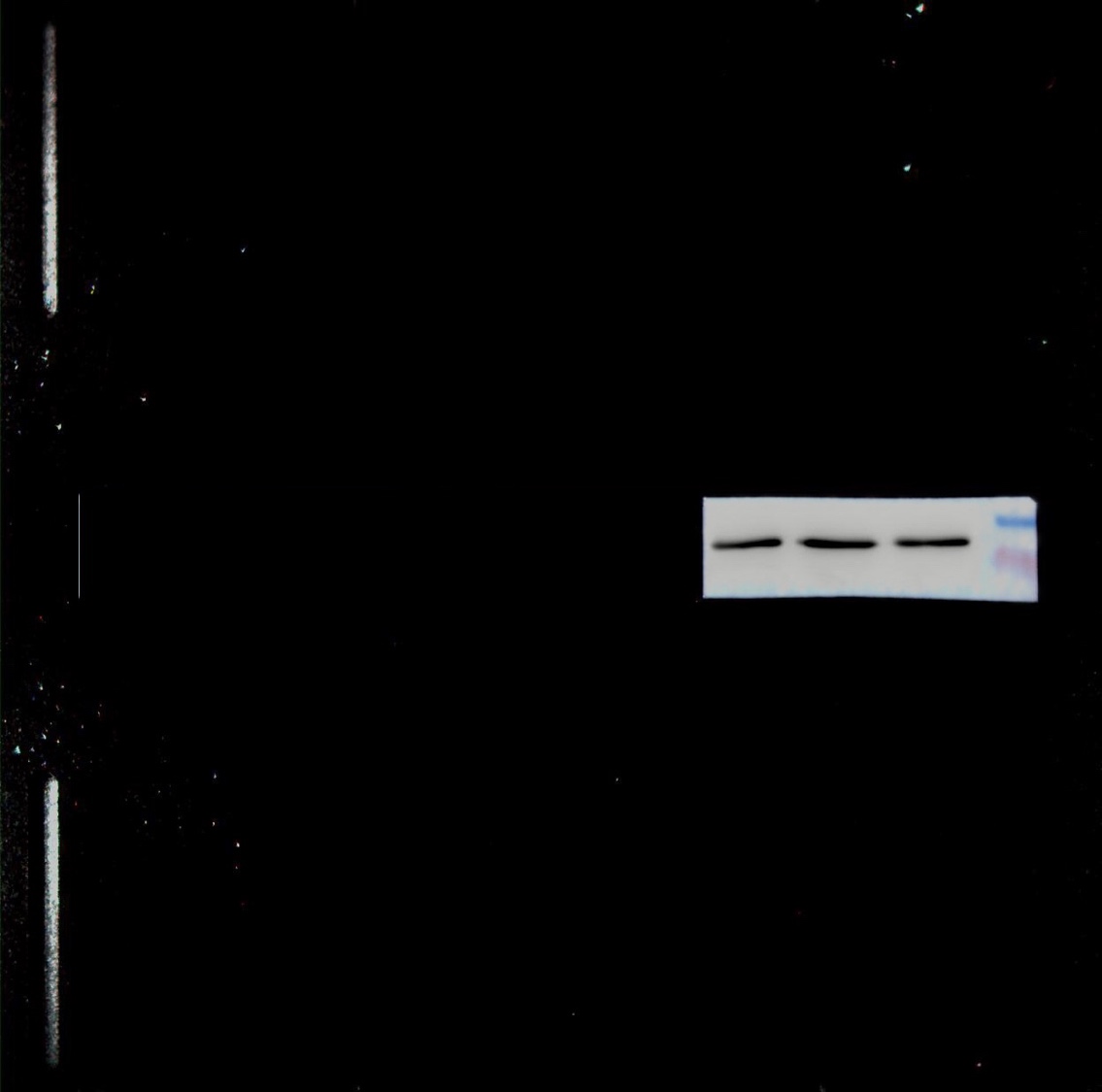


GAPDH-LoVo


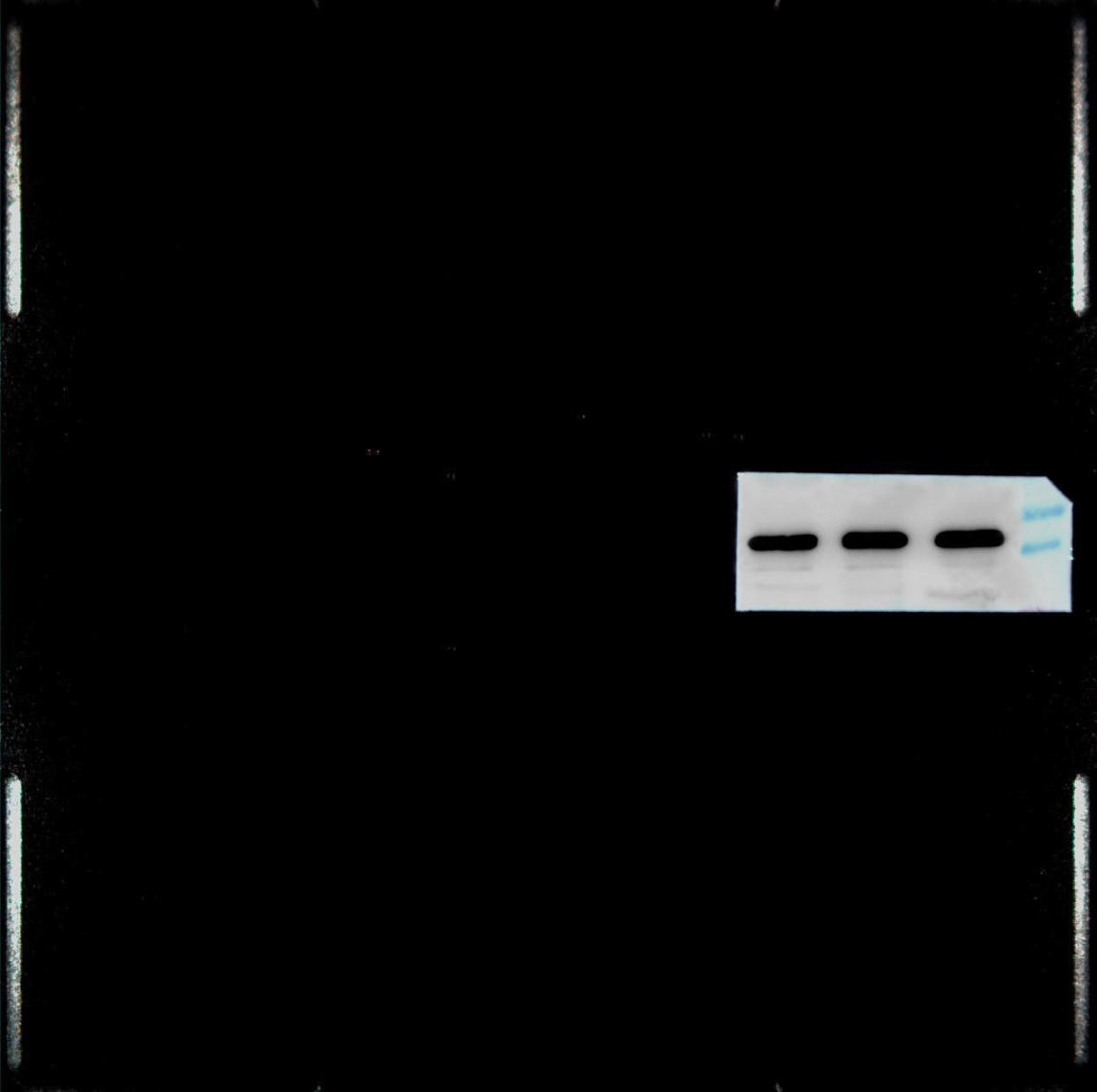


**Figure 3E**

HMGA2


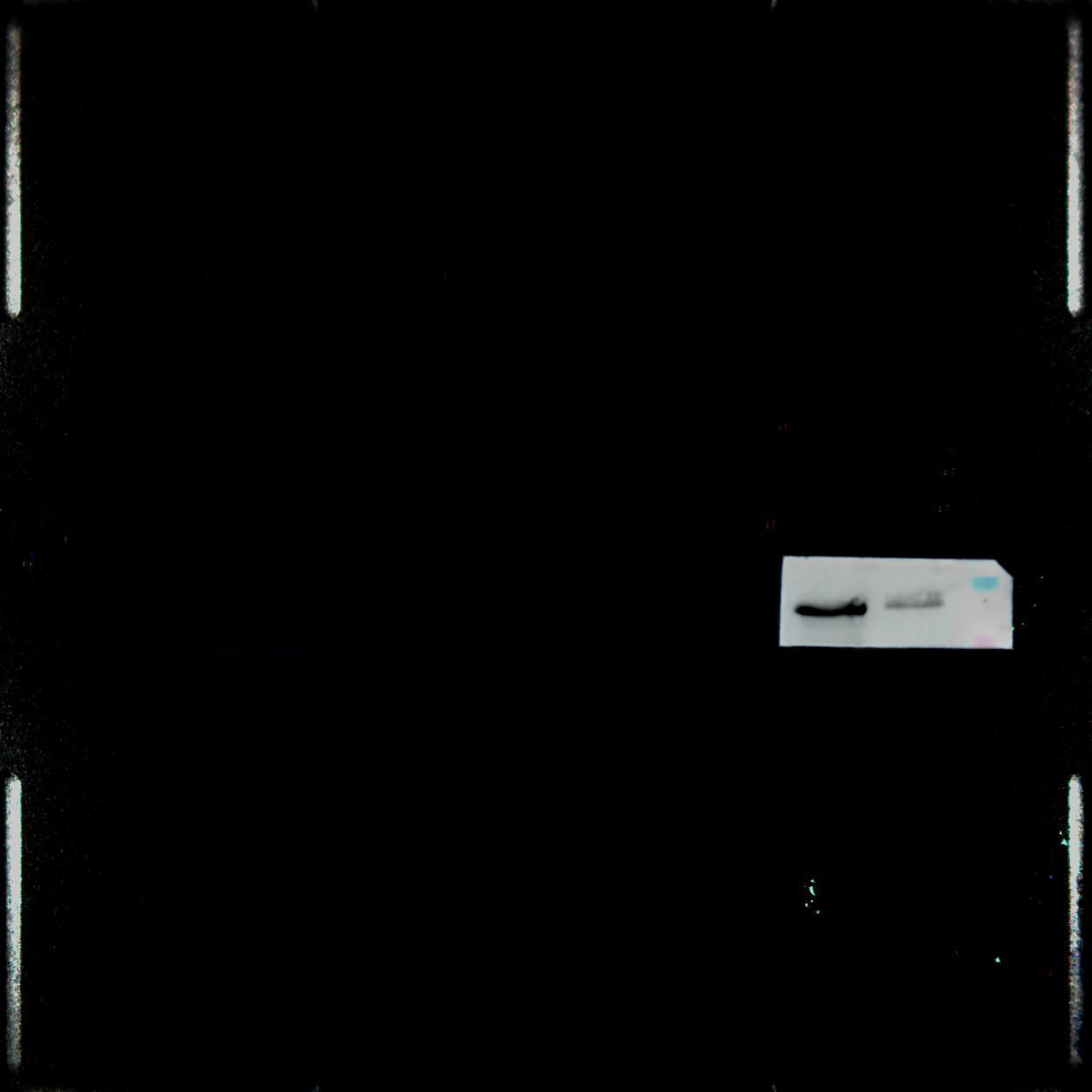


GAPDH


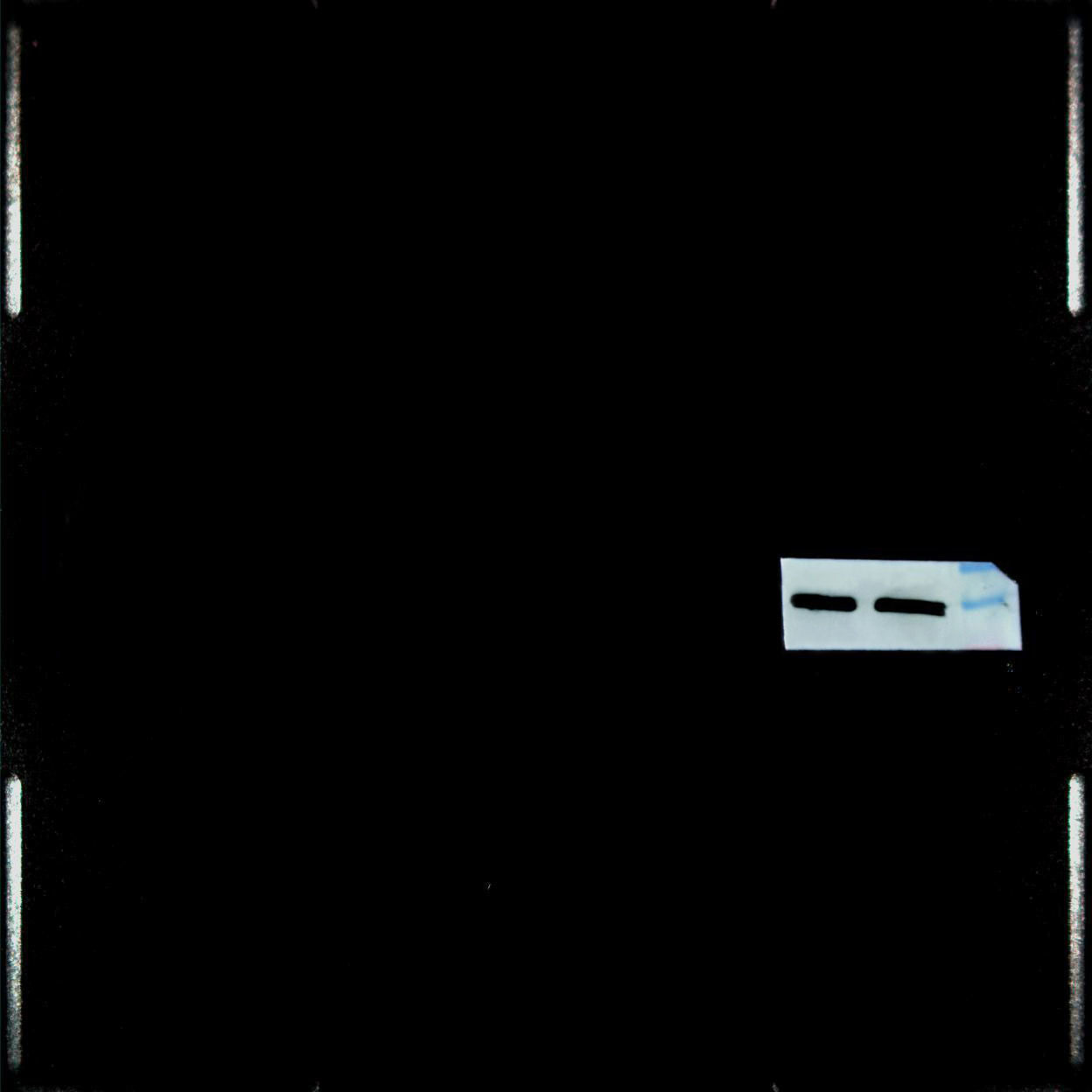


**Figure 4D**

KDM4C


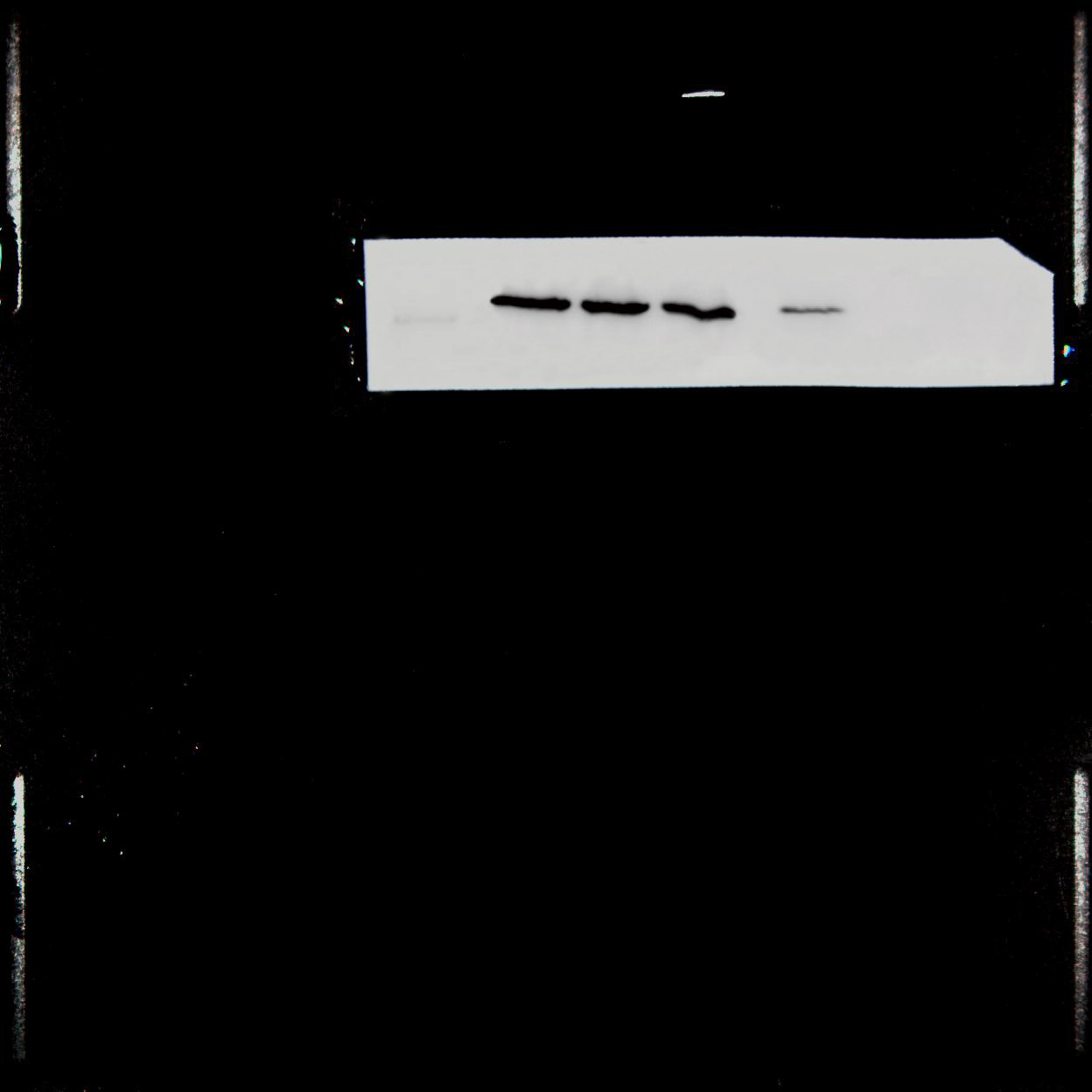


**Figure 4K**

HMGA2


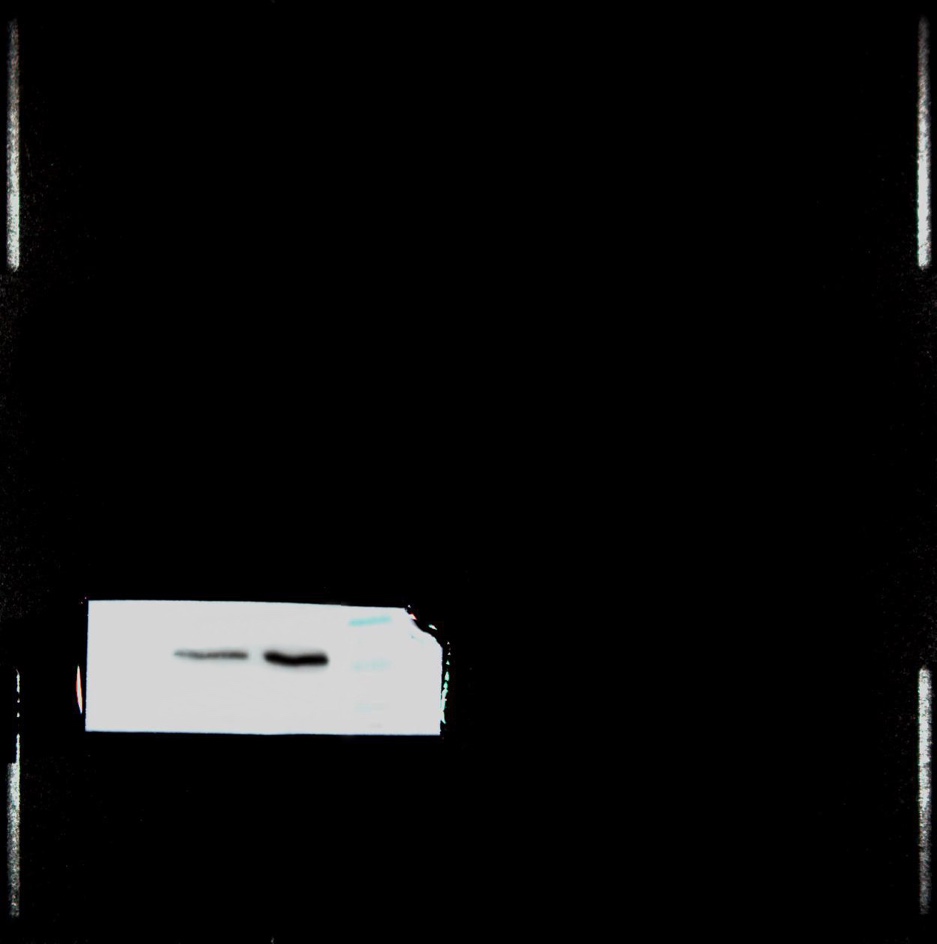


GAPDH


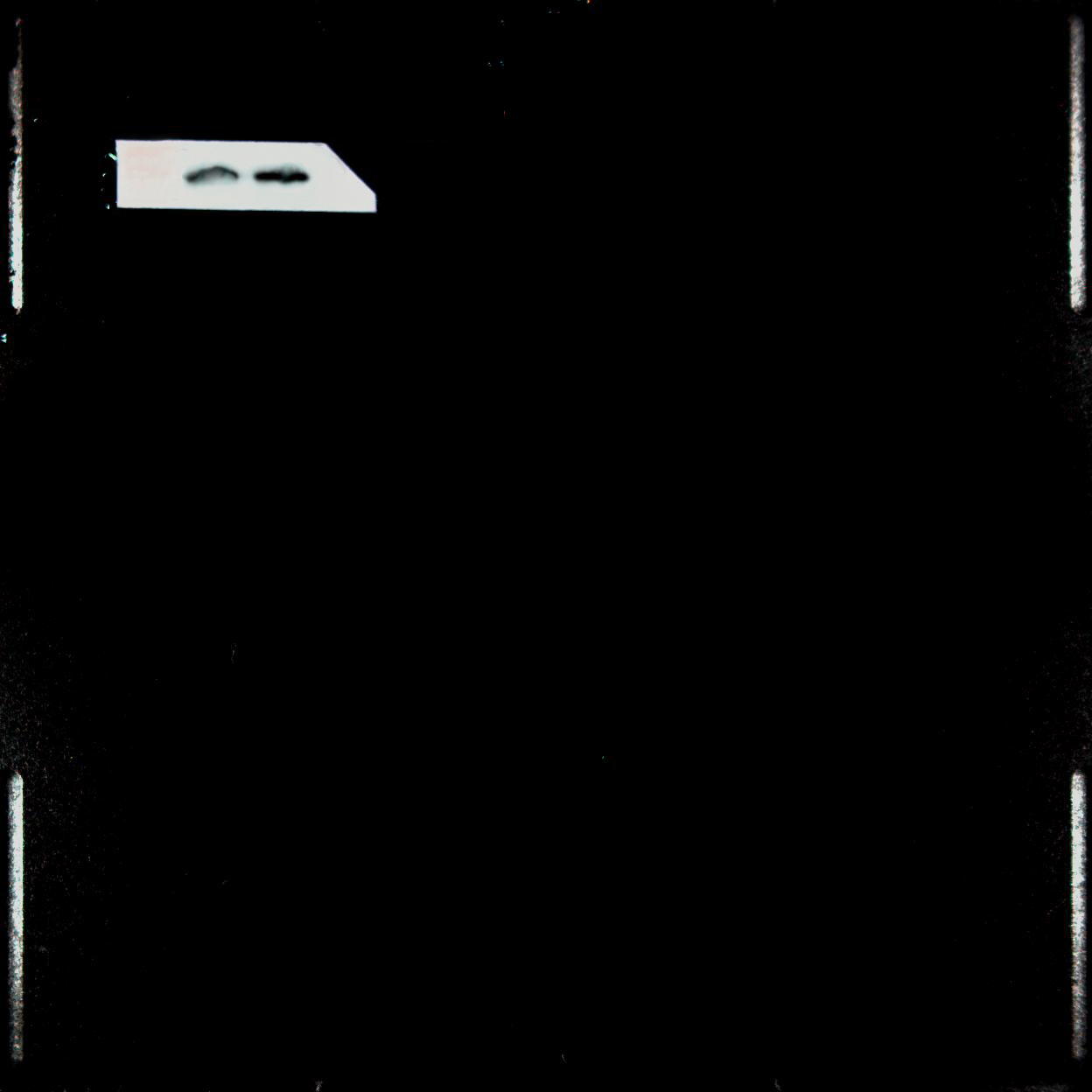


**Figure 6I**

ADAM19


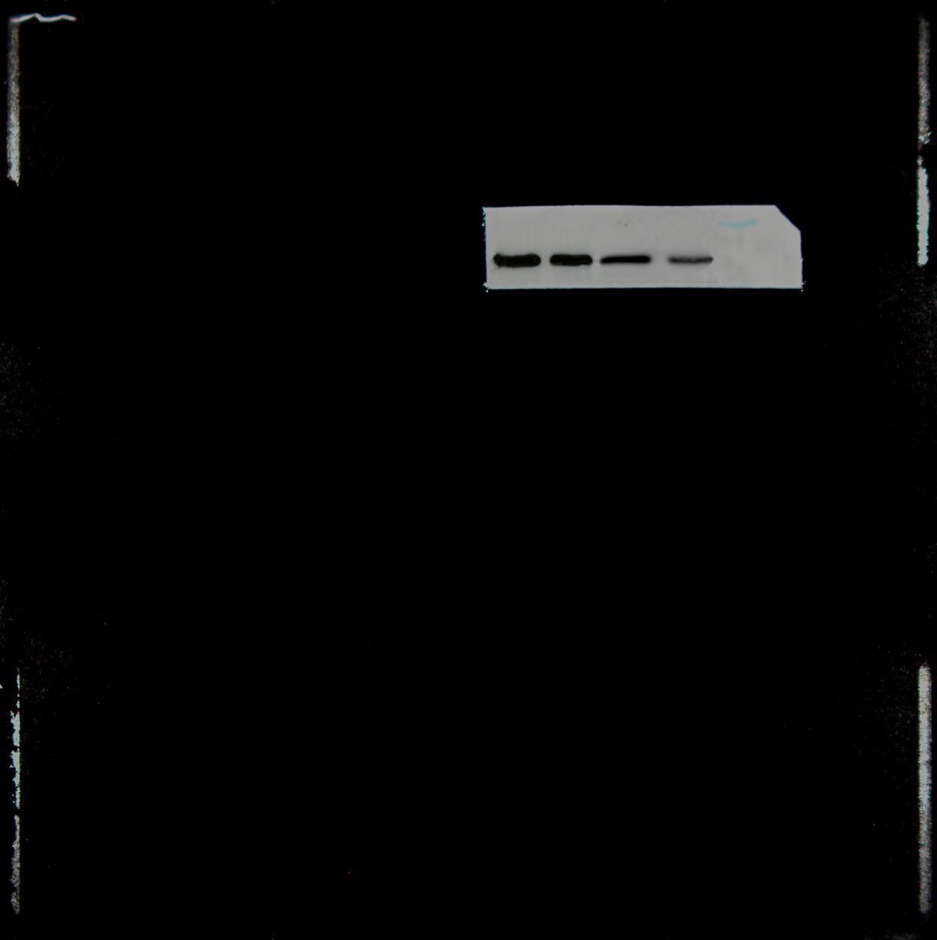


BMP4


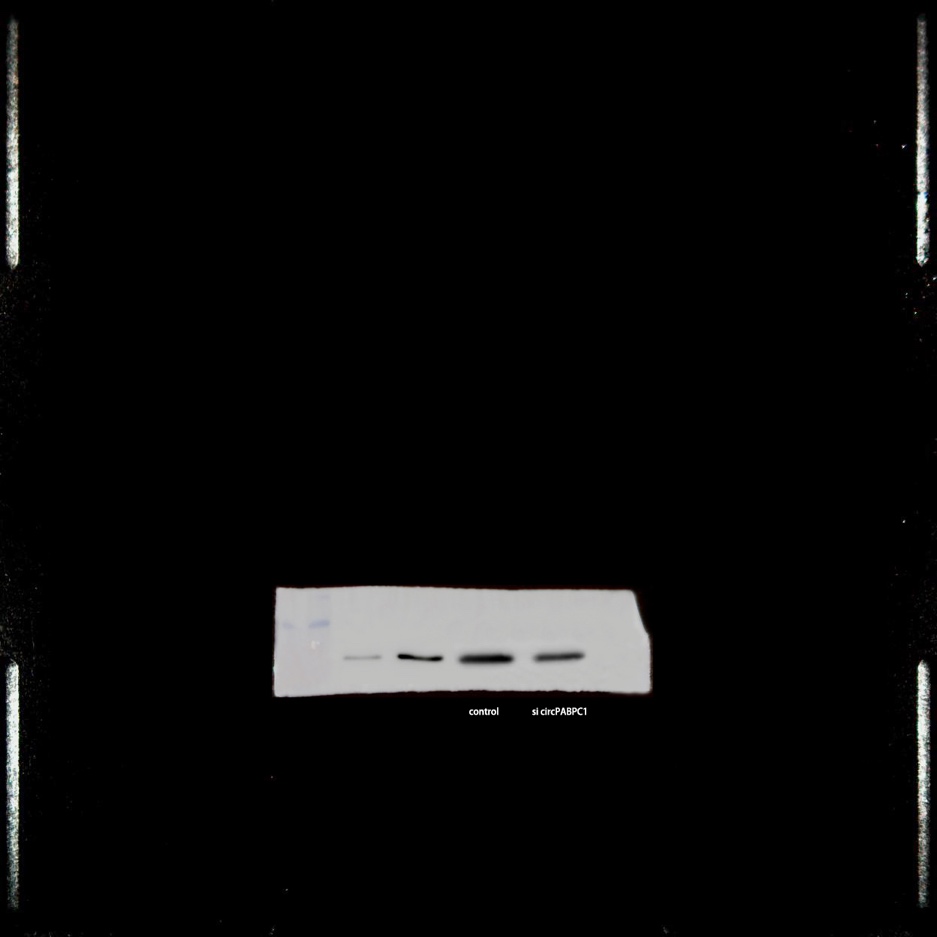


GAPDH


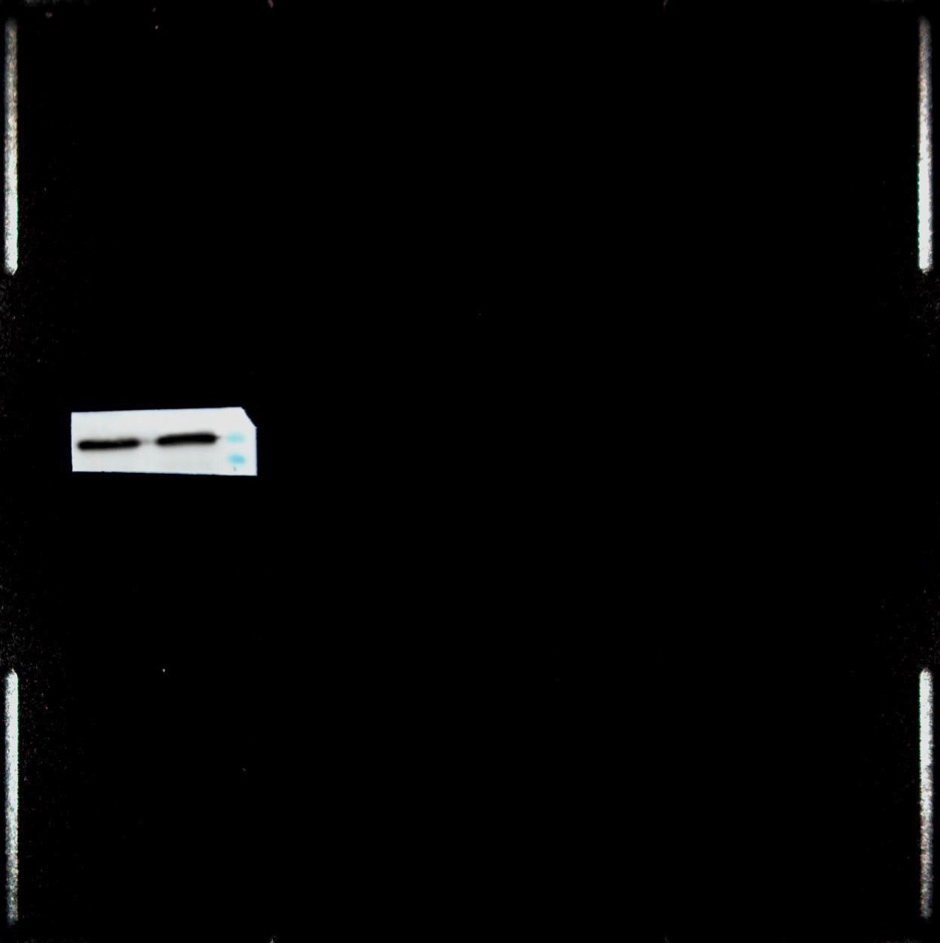

Supplement: Supplementary file 2 — Supplemental materials-Original blot [file 41420_2022_1124_MOESM2_ESM.docx]
